# Beyond Everyday Small Talk: A Qualitative Study on Registered Nurses' Confidential Conversations in Palliative Care

**DOI:** 10.1111/jan.17098

**Published:** 2025-06-03

**Authors:** Tove Stenman, Ylva Rönngren, Ulla Näppä, Christina Melin Johansson

**Affiliations:** ^1^ Department of Health Sciences; Nursing Science Mid Sweden University Sundsvall Sweden

**Keywords:** confidential conversations, focus groups, nurse, nurse–patient relations, nursing, palliative care, professional development, professional education, reflective practice

## Abstract

**Aim:**

To explore and gain a deeper understanding of how registered nurses in palliative care develop personal and professional approaches in confidential conversations with patients.

**Design:**

A qualitative study using focus groups.

**Method:**

Between March and May 2024, 22 registered nurses working in specialised palliative care in the northern region of Sweden participated in five focus groups. The discussions were recorded, transcribed verbatim and analysed using interpretive description.

**Findings:**

Registered nurses pursued meaningful, supportive interactions during confidential conversations. Their reflections revealed vulnerabilities and the importance of continuous self‐reflection, fostering growth, resilience and professional development. They sought ways to process emotional challenges, from individual reflection to peer discussions and structured supervision, refining their approaches. Four themes emerged: balancing external demands with inner motivation, recognising personal limitations, managing compassion with professional responsibility and gradually building trust.

**Conclusion:**

Reflection and continuous professional development are essential for navigating confidential conversations in palliative care. These practices help registered nurses balance empathy with boundaries whilst managing emotional and professional challenges. Peer support and shared learning, as well as fostering self‐awareness and emotional resilience can enhance care quality and promote sustained professional growth across healthcare settings.

**Implications for the Profession and/or Patient Care:**

This study highlights the emotional challenges registered nurses face in confidential conversations with patients at the end of life. Reflection and support help them handle these challenges and promote person‐centred care by enabling patients to express their inner thoughts and wishes. The findings apply to palliative care and other settings caring for patients at the end of life.

**Reporting Method:**

Findings were reported following the Consolidated Criteria for Reporting Qualitative Research guidelines.

**Patient or Public Contribution:**

This study did not include patient or public involvement in its design, conduct or reporting.

**Abbreviation**
RN: registered nurse

**Summary**

- What does this paper contribute to the wider global clinical community?
  ○ This study enhances understanding of the emotional challenges faced by registered nurses during confidential conversations with patients at the end of life.
  ○ Registered nurses, whilst balancing empathy with professional responsibility, require opportunities for reflection and peer support to manage these challenges effectively.
  ○ Providing registered nurses with structured support enables them to strengthen their resilience, enhance the quality of care and ensure patients can express their needs and wishes in these critical conversations.

## 1 Introduction

In palliative care, *confidential conversations* refer to intimate exchanges where patients disclose personal, existential issues and reflections on life and death (Stenman et al. 2023, 2024). These conversations often address sensitive topics related to end‐of‐life experiences and provide emotional support whilst helping patients process existential concerns (Tarbi et al. 2021; Stenman et al. 2024).

Everyday small talk between registered nurses (RNs) and patients can naturally evolve into such confidential conversations (Macdonald 2016; Tarbi et al. 2021). Research indicates that these conversations are typically initiated by patients, reflecting their awareness of life's imminent end and a desire to share personal stories (Tarbi et al. 2021; Stenman et al. 2023, 2024). Despite their importance, confidential conversations are described as challenging for RNs due to their unpredictability and emotional intensity (Stenman et al. 2023). RNs must handle this uncertainty to provide support during vulnerable moments, making confidential conversations both complex and emotionally demanding (Tarbi et al. 2021).

Patients with palliative care needs live with life‐threatening or life‐limiting illnesses or are of advanced age. Their needs span the entire palliative care journey, from early to late stages. Palliative care aims to relieve suffering, enhance quality of life and provide holistic, person‐centred support (Payne et al. 2022). Each year, around 56.8 million people worldwide, including 25.7 million in their final year of life, have palliative care needs. As populations age and noncommunicable diseases become more common, this need is expected to rise (World Health Organisation [WHO] 2020). Patients with palliative care needs are found across various settings—hospitals, home care, hospice and consultation service. Regardless of the care setting, the focus extends beyond just disease treatment and includes emotional and existential well‐being (Payne et al. 2022).

This study aims to deepen the understanding of how RNs in palliative care develop personal and professional approaches to conducting confidential conversations with patients, with a particular focus on how reflective practices and continuous professional development contribute to their communication skills and emotional resilience.

## 2 Background

The inevitability of death is universal yet confronting it can be emotionally challenging for patients and caregivers (Croker et al. 2022). In palliative care, RNs support patients facing limited time due to illness or advanced age, addressing physical, social, emotional and existential needs (World Health Organisation [WHO] 2020). The awareness of their limited time left in life can cause existential distress, highlighting the need for tailored conversations to alleviate suffering and offer support (Hussain 2020).

According to Oberle and Davies (1993), core elements of palliative nursing include presence, listening and creating shared meaning—all of which rely on effective communication. Through communication, RNs respond not only to patients' physical needs but also to their emotional, social and psychological concerns (Oberle and Davies 1993). Various types of conversations are identified in caring for patients with palliative care needs, such as advanced care planning (Bigger et al. 2023) and serious illness conversations (Bernacki et al. 2015). Of particular interest are those that occur spontaneously (Tarbi et al. 2021), described as everyday small talk (Macdonald 2016). Such casual exchanges can evolve into more confidential conversations (Stenman et al. 2023).

These conversations, central to person‐centred care, require professionalism, respect and attentive listening (Ohlén and Friberg 2023). Confidential conversations can offer patients a sense of respite and relief. If their needs for support remain unmet, they may feel unseen, which can deepen their suffering. Patients' preferences for conversation vary over time; flexibility and active listening are essential (Stenman et al. 2024). RNs describe confidential conversations as their responsibility to recognise and respond to (Stenman et al. 2023).

Although emotionally challenging for both patients and caregivers, such conversations are essential to providing adequate support for patients with palliative care needs (Croker et al. 2022). Managing them requires professional knowledge, adaptability and awareness of personal suffering. Meaningful dialogue fosters engagement and alleviates distress, promoting well‐being (Croker et al. 2022; Ohlén and Friberg 2023). Continuous learning enables RNs to develop a professional identity rooted in compassion, enhancing job satisfaction and improving patient care (Cao et al. 2023).

Compassion, an innate response to patient suffering, linked to empathy and altruism, is fundamental to compassionate care, but can be strained by overwhelming distress or perceived futility (Arman 2023). RNs may feel powerless, stressed or frustrated, affecting their ability to support patients (Portoghese et al. 2020; Rattner 2019), leading to variations in the support provided (Croker et al. 2022). Limited resources and inadequate support further challenge resilience, highlighting the need for strategies to sustain presence and validate patients' experiences (Rattner 2019).

Despite these challenges, finding meaning in their work can provide RNs with recovery and fulfilment. Helping others enhances job satisfaction; existential conversations are described as enriching, reducing emotional exhaustion whilst bolstering confidence (Baqeas et al. 2021). Supportive work environments promoting reflection and dialogue empower RNs to handle emotional challenges, sustaining their well‐being and professional development (Baqeas et al. 2021; Portoghese et al. 2020).

Although these interactions are recognised as important, there is limited understanding of how RNs develop the personal and professional approaches needed to manage such conversations (Ohlén and Friberg 2023). Whilst these conversations offer opportunities for relationship‐building and skill development, the specific processes through which RNs refine their communication and handle emotional challenges remain underexplored (Croker et al. 2022).

## 3 The Study

### 3.1 Aim

To explore and gain a deeper understanding of how RNs in palliative care develop personal and professional approaches in confidential conversations with patients.

### 3.2 Design and Theoretical Framework

This study is grounded in social constructivism, which views knowledge as co‐created through social interaction and shared meaning (Patton 2015). This epistemological perspective aligns with the study's focus on understanding how RNs develop personal and professional approaches in confidential conversations. Social constructivism provided a foundation for exploring how meaning is negotiated and shared amongst participants, influencing both the research design and analysis.

To derive insights aligned with nursing's epistemological principles, interpretive description (S. E. Thorne 2016) was employed. This methodological approach is well suited for applied healthcare research as it enables the exploration of complex, practice‐based phenomena whilst maintaining a strong connection to clinical relevance. A qualitative research design using focus groups was chosen to facilitate an in‐depth exploration of participants' experiences. Focus groups provide a dynamic setting where participants can challenge, refine and expand each other's perspectives, leading to richer data generation (Krueger and Casey 2015).

Person‐centred care highlights the importance of seeing the patient as a unique individual with their own experiences, values and preferences (Ohlén and Friberg 2023). This aligns with the social constructivist view, where meaning is shaped through relationships and context. Oberle and Davies (1993) adds relevant concepts such as presence, listening and shared meaning—all central to confidential conversations. Together, these frameworks may interact to provide a more nuanced illumination of relational and reflective communication patterns in palliative care.

In presenting the study, we adhered to the Consolidated Criteria for Reporting Qualitative Research (COREQ), a 32‐item checklist developed by Tong et al. (2007; Appendix S1).

### 3.3 Study Setting and Recruitment

Palliative care in Sweden is provided across hospitals, hospices, nursing homes and patients' homes, with access based on need rather than prognosis. This enables early referral for symptom management and psychosocial support at various stages of illness (Payne et al. 2022). The study was conducted in specialised palliative care units in the North Region of Sweden, including hospices, hospital wards with round‐the‐clock care and home healthcare teams. This sparsely populated region, with around 900,000 residents (Statistics Sweden 2021), encompasses rural and urban areas, highlighting the diverse contexts of palliative care delivery.

Purposeful sampling (Patton 2015) was employed to select the participants. Information letters about the study were distributed to the administrative managers of five nearby specialised palliative care units. Four managers, representing two hospices and two home health care teams, agreed to participate and disseminate written study information to RNs in their units. The inclusion criteria specified that participants had to be RNs employed in specialised palliative care. The RNs received written information about the study and were invited to participate. Personal contact via e‐mail was established by the first author (T.S.) with the RNs before the focus groups, during which they received further information. The focus groups were scheduled during working hours in quiet settings to minimise disturbances.

The research team comprised four RNs with expertise in qualitative research, palliative‐ and psychiatric care. Three researchers (Y.R., U.N., C.M.J.) hold a PhD in nursing, whilst the first author (T.S.) is a PhD student with clinical experience in palliative care. Their backgrounds informed the study whilst requiring reflexivity to minimise preconceptions.

### 3.4 Data Collection

Between March and May 2024, 22 RNs participated in five focus groups, see Table 1. Each group had three to five participants, with one dropout from focus group 3 due to workload. For an overview of the focus group sessions, see Table 2. The focus groups were conducted following Krueger and Casey's (2015) guidelines, ensuring rigour and consistency. The ideal group size is three to eight participants. Whilst all participants worked in specialised palliative care, they varied in gender, nursing background and experience, allowing for both shared and diverse perspectives. Each group consisted of staff from the same unit, where workplace familiarity facilitated connection and idea exchange (Krueger and Casey 2015). Participants were also accustomed to group discussions in their clinical practice. The focus group sessions were held at the participants' own workplaces, in designated and undisturbed rooms.

**TABLE 1:** Demographic data of the participants in the focus groups, *N* = 22.

	*n*	%	Focus group 1 *n* = 4	Focus group 2 *n* = 4	Focus group 3 *n* = 3	Focus group 4 *n* = 6	Focus group 5 *n* = 5
**Gender**							
Female/male/other	19/3/0	85/15/0	4/0/0	3/1/0	2/1/0	5/1/0	5/0/0
Age	23–67 (Mdn 46.5)		35–62 (Mdn 49.5)	41–67 (Mdn 50.5)	47–63 (Mdn 53)	23–55 (Mdn 34)	38–60 (Mdn 43)
Years working as a registered nurse	1–36 (Mdn 12)		12–36 (Mdn 22)	7–27 (Mdn 22)	19–24 (Mdn 22)	1–32 (Mdn 6.5)	1–11 (Mdn 10)
Years working in palliative care	0–24 (Mdn 6)		4–24 (Mdn 9.5)	4–18 (Mdn 8)	10–16 (Mdn 15)	0–18 (Mdn 3)	1–9 (Mdn 6)
**Care settings**							
Inpatient ward/home healthcare	11/11	50/50	4/0	4/0	3/0	0/6	0/5
**Education**							
Graduate without further education/graduate with further education	10/12	40/60	0/4	0/4	1/2	6/0	3/2

**TABLE 2:** Overview of focus group sessions, *N* = 5.

Focus group	Date	Participants *N* = 22	Place for interview	Drop‐out	Moderator	Assistant	Duration
1	2024‐03	4	On the unit	0	C.M.J.	T.S.	1 h 13 min
2	2024‐03	4	On the unit	0	C.M.J.	T.S.	1 h 23 min
3	2024‐04	3	Teams	1	T.S.	Y.R.	1 h 30 min
4	2024‐05	6	On the unit	0	T.S.	Y.R.	1 h 10 min
5	2024‐05	5	On the unit	0	T.S.	Y.R.	1 h 34 min

Participants were verbally briefed on the study's purpose, voluntariness and right to withdraw and provided written consent before the sessions. The moderator and assistant introduced themselves and outlined the focus group guidelines to ensure open discussion. This approach aimed to minimise the risk of perpetuating mistrust, particularly if participants felt uneasy speaking in front of colleagues or perceived power imbalances. Instead, the goal was to foster familiarity and trust (Krueger and Casey 2015).

The participants completed background information forms and were asked to review a patient case (Appendix S2) before the sessions, serving as an icebreaker and discussion guide. The use of a patient case helped establish a shared understanding of what constitutes a confidential conversation, offering a concrete and relatable reference point to guide both discussion and analysis within the focus groups. The focus groups followed the structure outlined by Krueger and Casey (2015; Appendix S3). The conversation guide was designed to explore participants' experiences, emotions and perceived challenges in conducting confidential conversations. The focus group questions were organised:

- Opening and introductory questions: Initial reflections on the patient case and general experiences with confidential conversations.
- Exploratory questions: Feelings during such conversations, perceived success in connecting with patients and opportunities and obstacles.
- Key questions: Support needed to facilitate confidential conversations, including knowledge, tools and organisational structures.
- Final reflections: Ensuring patients have opportunities to engage in conversations and any additional insights from participants.

A pilot focus group with experienced RNs, but without experience in specialised palliative care (an inclusion criterion), was conducted and subsequently excluded from the analysis.

In qualitative research, data saturation is of limited value since capturing all experiences and perspectives is impossible. Instead, the emphasis should be on the insights that emerge from the data, prioritising participants who can offer a deeper understanding of the phenomenon (S. Thorne 2020).

All sessions were digitally recorded and transcribed verbatim to enable comprehensive analysis. Participants were invited to review their transcripts; however, all declined. For details on data storage and confidentiality, see the Ethical Considerations (3.7).

### 3.5 Data Analysis

Using S. E. Thorne's (2016) interpretive description, the data were systematically analysed to identify themes and insights with practical implications for nursing practice. This approach prioritised understanding the unique perspectives of RNs, aiming to generate insights relevant to both practice and research, grounded in nursing's epistemological foundations. Although notes were taken by the assistant during each focus group session, they were excluded from the analysis.

S. E. Thorne (2016) stressed the importance of systematic rigour in the analysis, ensuring that the phenomenon under study was thoroughly examined and contextualised within its broader framework. The analysis was guided by two central questions: ‘What has happened?’ and ‘What can we learn?’ The transcriptions were reviewed several times, with key sections highlighted for more in‐depth analysis. Initially, key comments were identified and coded inductively, allowing patterns and categories to emerge from the data. The codes, representing units of meaning, were derived from these statements and grouped based on similarities and differences. Through an iterative process, broader conceptual categories emerged, capturing recurring patterns and relationships. The first author (T.S.) organised these categories, which were reviewed, summarised and refined into preliminary themes through research team discussions. The focus group analysis aimed to integrate diverse experiences, track shifts in opinion, present logical arguments and develop coherent themes. The key criteria for categorisation included the variety and emotional intensity of comments, their specificity and the emphasis on certain remarks (Krueger and Casey 2015). See Appendix S4 for examples of this process.

### 3.6 Rigour and Reflexivity

Rigour was maintained using S. E. Thorne's (2016) quality criteria. Epistemological integrity was ensured through co‐authors' feedback, and representative credibility was enhanced by including diverse nursing experiences. An audit trail supported reflection on the dataset, and verbatim transcripts ensured interpretive authority. Regular meetings facilitated coding discussions (S. E. Thorne 2016).

The research team reflected critically on their positionality and reflexivity throughout data collection and analysis. As experienced RNs in palliative and psychiatric care, the team acknowledged pre‐existing understandings and considered the familiarity between some participants and the first author (T.S.) to ensure transparency. Memo writing captured emerging insights and reflections, helping the team to ensure the analysis reflected participants' experiences rather than researchers' preconceptions (Patton 2015).

### 3.7 Ethical Considerations

Ethical approval for the study was granted by the Swedish Ethical Review Authority (Dnr 2024‐00341‐02), following the guidelines of the World Medical Association Declaration of Helsinki (2024).

All participating RNs received written and verbal information about the study, and administrative managers signed agreements allowing their participation. Each RN provided informed consent, understanding that participation was voluntary and they could withdraw at any time during the focus group without explanation. If any RN experienced strong emotions during the interview, the moderator and assistant first offered support, and if needed, the unit manager would be contacted for further assistance. The researchers took responsibility for ensuring participants felt supported throughout the process.

Transcripts were stored on a secure, password‐protected computer accessible only to the research team. A separate coded list was maintained to ensure participant anonymity. All data handling followed ethical standards and best research practices (World Medical Association Declaration of Helsinki 2024).

### 3.8 Findings

**FIGURE 1:**
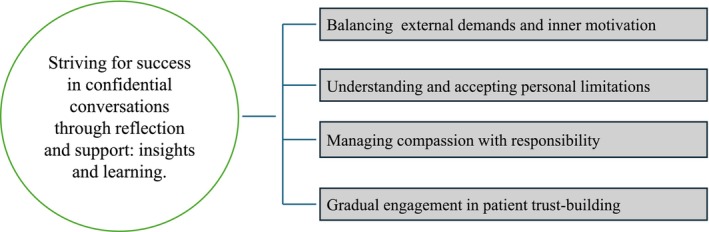
Interconnected themes: RNs' development in confidential conversations within palliative care.

This study aimed to explore how RNs in specialised palliative care develop their personal and professional approaches in confidential conversations with patients. An overarching theme was identified: striving for success in confidential conversations through reflection and support: insights and learning. The interconnected themes revealed through the analysis are presented in Figure 1, with each theme supported by relevant quotes from participants, which are based on the analysis. These quotes illustrate the findings and are referenced by the corresponding focus groups (1–5).

In confidential conversations with patients, RNs were guided by a strong desire to foster meaningful and supportive interactions. The analysis revealed a consistent pattern: when they felt unable to connect as hoped or they experienced a deep emotional impact, a sense of inadequacy could lead to moments of doubt and self‐reflection. This interplay highlights their vulnerability and the role of ongoing self‐reflection to foster growth, resilience and professional development. RNs actively sought opportunities to process these experiences, whether individually, through informal discussions with colleagues or within structured supervision. These reflective practices were pivotal in refining their personal and professional approaches.

Reflection helped RNs handle the emotional and professional challenges inherent in confidential conversations. Four themes emerged from their reflections, illustrating the diverse and often conflicting demands that they experienced. *Balancing external demands and inner motivation* reflected the challenge of meeting professional expectations with their internal motivation, often feeling pressured to handle confidential conversations. This interplay motivated them to succeed whilst confronting the realities of failure. *Understanding and accepting personal limitations* enabled the RNs to come to terms with their constraints, gaining a clearer understanding of their role and humanity. *Managing compassion with responsibility* highlighted the delicate balance between patient care and their own emotional well‐being, underscoring the necessity of personal compassion whilst maintaining professional distance. Finally, *gradual engagement in patient trust‐building* emphasised the importance of patience—a deliberate, ongoing process of developing trust with patients and recognising the value of small successes.

#### 3.8.1 Balancing External Demands and Inner Motivation

RNs described the challenge of balancing external demands, such as professional expectations, with their internal motivation to make a meaningful impact on patients' lives. This tension created a sense of pressure, because success or failure often depended on how well they could meet personal and professional standards. Focus group 1 discussed the pressures arising from these challenges:

RN 101: In the conversation, you get access to their innermost thoughts, I think. At least, that's the feeling you get.

RN 102: …it's really special… it makes you happy because it affirms that you're being trusted with something so important. But at the same time, you think

‘Wow, what do they expect of me now? Should I say something?’ It creates a kind of pressure… even as it's something beautiful. I try to just listen and respond quietly… because otherwise, you might say the wrong thing. So it does add some pressure, I think…

RN 104: Does it always feel like pressure?

RN 102: No, it depends… I just want to make sure I receive it well… to really honour it, and that's where the pressure comes in. And then you also wonder, “What should I do with this? Should this become our… moment to talk about it? Or… where should I go with it now?

This sense of pressure, combined with the fear of missing the moment or saying the wrong thing, made the interaction feel even more important. RNs questioned whether this was their only chance to connect meaningfully with the patient, adding further complexity to the task of balancing external expectations with their inner motivation. Reflecting on these experiences allowed them to handle these tensions and understand that balancing these demands was a crucial part of their development and practice.

When RNs perceived themselves as having missed an opportunity, feelings of inadequacy arose, prompting reflection on how to adjust their future approach. They recognised that not every conversation would meet both internal motivations and external expectations but that learning from these moments helped improve their practice. Success was often tied to allowing conversations to unfold naturally, without the pressure of specific outcomes. Reflection helped them understand that being present and empathetic was often enough, even if the conversation did not go as planned.

The balance between internal motivation and external demands was an ongoing challenge. When RNs felt powerless to guide a conversation as intended, they reflected on how to better manage this tension in the future. By intentionally creating space for reflection—individually, with colleagues or through supervision—they could better handle the complexities of balancing these competing demands. This process empowered RNs to feel more confident in their roles, knowing that resilience and compassion were key to managing internal and external pressures.

#### 3.8.2 Understanding and Accepting Personal Limitations

RNs recognised the importance of acknowledging and accepting their limitations. They realised that their ability to provide compassionate care and support was influenced by life circumstances, such as having a bad day, which impacted their ability to be fully present. This awareness highlighted the need to avoid striving for unattainable perfection and to recognise that sometimes ‘good enough’ was sufficient. RNs learned to be honest with themselves and accept that not always feeling sufficient in their roles was inevitable. This self‐awareness was crucial to their emotional well‐being, as illustrated by participants in focus group 4, who emphasised the importance of recognising their limits and stepping back.

RN 403: It's important to also look inward and recognise when, for example, you feel you can't be with a patient anymore… because it's very draining… because we deal with heavy issues, and there's always a risk we won't be able to cope in the long run… and truly surrender to that feeling… because sometimes we think we should just push through, not focus too much on how we feel… but sometimes, we need to surrender and acknowledge our limitations.

RN 404: I also think… that you need to think about yourself a bit.

RN 403: … and really give in to that feeling… because sometimes you need to be a bit kind to yourself and not feel ashamed… but dare to surrender and ask for help… there are others…

RN 405: Yes. We have the opportunity, it's just foolish not to acknowledge how you feel and ask for help, I think…

RNs experienced a tension between their desire to help and the reality that some situations could not be changed. Although they strove to offer comfort, feelings of inadequacy arose when they were unable to solve the patient's problems. Over time, they understood that sometimes, simply ‘being there’ provided the most meaningful support. Through experience, RNs developed a deeper understanding of their limitations, which allowed them to navigate challenging situations more effectively. They learned to prioritise presence over problem‐solving and to reflect on questions such as, ‘Who am I having this conversation for?’

Through reflection and peer support, RNs gained insight into their limits. This was emphasised in focus group 5, where participants discussed how these processes helped them better understand their own boundaries.

RN 503: We spend so much time together… so we get to know each other in a way that few colleagues do… especially since we also share these kinds of things together…

RN 505: So everyone… who works here knows the boundaries we operate within… the terrible things that can happen in life… I'm thinking, you who are quite new… [turns to RN 507] what kind of support is most important for you, for example?

RN 507: Well, it's the colleagues…

RN 505: Being able to share?

RN 507: Yes, it's also about hearing others' experiences… then my experience doesn't seem so strange.

RN 506: …What I think helps the most is getting good guidance on how to shift focus… focus on the conversation and move it outside of yourself. It's not about what I can do, or what I can solve, or what I can contribute with, really… It's more about just being there…

Support from colleagues who understood the challenges of their roles was particularly valuable. By reflecting with their colleagues to understand and accept personal and professional limitations, RNs fostered individual and collective development. Embracing their humanity and engaging in reflective practice, They cultivated a supportive network that benefited themselves and the patients.

#### 3.8.3 Managing Compassion With Responsibility

Empathy and compassion were crucial in confidential conversations, requiring RNs to balance personal connection with professional distance. Compassion fostered trust, enabling patients to feel acknowledged, valued and understood. However, deeply experiencing patients' suffering demanded high levels of self‐awareness and emotional management.

Connecting with a patient's suffering could be enriching since it provided a deep, transformative experience of emotional closeness, offering a sense of profound understanding. However, it also carried the risk of becoming burdensome or unbearable. RNs had to find a balance to remain present and empathetic without becoming strained. Mindful reflection served as a tool in this process, enabling them to maintain professional boundaries whilst nurturing compassion. Reflective practice supported RNs in approaching these emotionally charged moments with awareness rather than reactivity. As expressed by participants in focus group 5, these emotional responses often appeared through conversations with patients.

RN 504: I'm feeling very emotional as we sit here talking… When I think back to the tutorial, what stuck with me was something the deacon said. She told us, ‘You're used to seeing the peaceful, beautiful deaths, and those are the ones you strive for – but you also need to witness the most difficult, the ugliest deaths.’ No one should ever have to see that. At first, I felt selfish for even thinking about it, because it's far worse for the patient. But she articulated it perfectly… it was like I didn't even want to accept that someone could die that way. That was my feeling at the beginning.

RN 501: Yes, it feels incredibly unfair that anyone should go through that.

RN 503: And we were powerless… we tried and tried, but there was nothing we could do to change it…

Conversations could evoke feelings of inadequacy and helplessness, making emotional regulation essential for resilience. RNs strove to manage their emotions without being overwhelmed, even if that meant occasionally crying with the patient. They viewed crying and sadness as part of the human experience and essential forms of emotional expression—so long as these responses did not overshadow the patient's own experience.

Sharing challenges with peers offered emotional relief, allowing RNs to process their feelings within a supportive network. This need for reflection and emotional processing was highlighted in focus group 2, when participants described how structured spaces for discussion helped them.

RN 202: I think it feels good… these small moments of reflection. It happens a lot—just micro‐reflecting with colleagues—but we also have our…

RN 204: Yes, it's important.

RN 202: structured reflective conversations. It's a relief to have a space where you can put things into words.

RN 205: Yes, exactly.

RN 202: That's how I felt when they confided in me and shared something difficult. You try to stay professional, but sometimes you just get affected. Then it's a relief to talk to someone—especially wise and supportive colleagues.

Through moments of humour and shared vulnerability, including tears, they found ways to create necessary emotional distance whilst maintaining their ability to care. Peer support was complemented by self‐care strategies, such as mindfulness, which some RNs found helpful in centring themselves and managing emotional stress. This combination of peer and personal support offered insights into their responses and strengthened their emotional resilience, allowing them to balance compassion with professional distance. By focusing on patients' needs whilst managing their own emotions, RNs could validate patients' experiences without becoming overwhelmed, ultimately supporting their well‐being and compassionate patient care.

#### 3.8.4 Gradual Engagement in Patient Trust‐Building

In their efforts to create the right conditions for confidential conversations, RNs reflected on the importance of gradual engagement in trust‐building. They emphasised that trust was neither immediate nor linear; it developed progressively, requiring patience and consistency. This process was shaped by RNs' ability to be present and attentive, allowing patients time and space to speak at their own pace. RNs recognised that confidential conversations often emerged spontaneously rather than through direct engagement, occurring during practical tasks where tensions were reduced.

RNs needed to be flexible and adapt to varied situations. They recognised that ‘good enough’ moments—where progress might be slow but still meaningful—were valuable in building trust. This reflection underscored that gradually developed trust positively affected the well‐being of the RN and the patient. Each small success, such as a patient sharing a personal story, was seen as an important investment in patients' well‐being. RNs also acknowledged that other colleagues sometimes succeeded in areas where they did not, fostering humility within the team. This sentiment was reflected in focus group 3.

RN 303: You have to be patient and give it time…

RN 302: Yes.

RN 301: Exactly.

RN 303: And sometimes it's also about not always getting to reap what you sow, but that… it's like a process… that it takes time, and eventually, the person is ready. Then it's the one who's there, who's receptive, and it lands in their lap… Sometimes it's simply that the time has come, and the person… wants to talk. And when that happens, it's the one who stays, who listens, and is right there in that moment, who gets it.

Through these reflections, RNs realised that patience and flexibility were key to building trust with patients. These qualities were central to their personal and professional development within confidential conversations. This reinforced the understanding that gradual engagement, rather than rushing towards immediate outcomes, was essential for creating conditions conducive to meaningful exchanges.

## 4 Discussion

This study aimed to explore and gain a deeper understanding of how RNs in palliative care develop personal and professional approaches in confidential conversations with patients. In confidential conversations with patients, RNs aimed to establish meaningful, supportive connections. However, challenges or emotional difficulties could lead to feelings of inadequacy and moments of self‐doubt, prompting reflection. RNs sought opportunities to process these experiences—individually, through informal discussions with colleagues or within structured supervision. These reflective practices appeared to support the development of their personal and professional approaches, which are prerequisites in nursing care for presence, listening and creating shared meaning (Oberle and Davies 1993), particularly in person‐centred conversations (Ohlén and Friberg 2023).

The findings highlight how reflective practices help RNs process emotions and better understand patients' needs—a foundation of person‐centred care (Ohlén and Friberg 2023). Such practices foster resilience, compassion and confidence in managing difficult conversations (Arman 2023). They are vital for handling emotional situations and essential to professional growth (Parola et al. 2018), enabling RNs to improve communication and navigate complex care (Croker et al. 2022). Continuous learning beyond formal education supports this balance, with informal settings like peer discussions and daily practice encouraging reflection, building confidence and easing stress (Pool et al. 2016).

RNs in this study described how reflection helped *balance external demands with inner motivation*, ultimately enhancing patients' well‐being and the quality of care provided. Reflective work environments support RNs in managing pressure, especially during difficult conversations (Guo et al. 2024). Strategies for aligning expectations with personal values are often closely linked to their commitment to providing supportive care (Oberle and Davies 1993). Peer discussions and daily practice fostered reflection, reduced stress and supported this balance (Pool et al. 2016). Supervision and collegial dialogue offered space to process emotions, gain perspective and strengthen strategies. These interactions enhance emotional stability and a sense of belonging, reinforcing mutual support (Kim and Chang 2022).

Another key finding was the value of recognising and accepting personal limitations. RNs who acknowledged their limits were better able to manage emotional reactions, supporting Kim and Chang's (2022) view that self‐awareness fosters stability and prevents overextension. Reflection, peer dialogue and supervision helped RNs navigate these limits and develop coping strategies. A culture of teamwork enabled them to lean on colleagues (Portoghese et al. 2020), enriching the supportive care framework through mutual presence and collaboration (Oberle and Davies 1993). These shared experiences not only ease emotional strain but also build the resilience needed for the complexities of palliative care (Mlambo et al. 2021).

The emotional challenges of *managing compassion with responsibility* were highlighted in this study. RNs reported sorrow, helplessness and distress when unable to alleviate patients´ suffering. Person‐centred care highlights the importance of understanding and respecting patients' emotional and psychological states to foster compassionate communication (Ohlén and Friberg 2023). As noted by Rattner (2022), RNs found themselves in an ‘in‐between space’ where they sought to relieve suffering without becoming emotionally overwhelmed. This balance between compassion and self‐care requires self‐reflection. RNs who handle these challenges sustain both emotional resilience and compassionate care. As Arman (2023) emphasised, establishing boundaries, combined with self‐reflection, is essential for managing intense empathy and preventing emotional exhaustion.

This emotional strain, particularly the feeling of powerlessness when unable to alleviate suffering, is well‐documented in previous research (Baqeas et al. 2021; Rattner 2022). Such experiences contribute to stress and, if unaddressed, can lead to burnout and exhaustion. RNs balance compassion with responsibility, manage emotional reactions, reduce frustration and sustain compassionate care (Cross 2019). Oberle and Davies's (1993) theory of supportive care deepens this understanding by highlighting the importance of reflection and emotional management, helping RNs align personal values with professional duties in sensitive, confidential conversations.

Finally, the *gradual process of building trust with patients* emerged as a fundamental component of confidential conversations. RNs recognised that trust could not be rushed and that patient‐centred, compassionate engagement was essential to fostering a therapeutic relationship. This requires sensitivity and awareness, as RNs need to assess each patient's readiness to engage and adjust their approach accordingly (Ohlén and Friberg 2023). As Parola et al. (2018) note, trust‐building creates a foundation for patients to share their emotions, strengthening the RN–patient bond and improving care. Oberle and Davies' (1993) deepens this understanding by highlighting the role of relational presence and shared meaning. These elements are central to person‐centred conversations, where trust and emotional connection are essential (Ohlén and Friberg 2023).

A work environment that supports peer interactions and provides structured supervision was crucial. The exchange of experiences within the team helped alleviate the emotional burdens and reinforced a shared understanding of the challenges. This support system allows RNs to handle difficult conversations with more confidence, fostering empathy and emotional stability (Peerboom et al. 2023). Supervision helps RNs process experiences, reflect on emotional responses and strengthen their ability to manage the emotional demands of care (Dobrina et al. 2020). Our findings showed that peer support and supervision not only supported emotional well‐being but also transformed difficult conversations into opportunities for professional growth. These systems enable RNs to manage the emotional complexities of confidential conversations, reducing the risk of emotional exhaustion whilst fostering resilience and enhancing the quality of care (Portoghese et al. 2020).

### 4.1 Methodological Discussion and Limitations

The qualitative design using focus groups was suitable for exploring RNs' reflections and approaches in confidential conversations with patients with palliative care needs. The group dynamic was beneficial as RNs were accustomed to speaking with each other and felt trust, fostering openness and enabling in‐depth sharing. A social constructivist approach facilitated collaborative knowledge creation, whilst the supportive atmosphere encouraged participants to express their thoughts, enriching the data (Krueger and Casey 2015).

Limitations were noted. The digital format of one focus group may have reduced spontaneity and participants' familiarity, potentially constraining critical views, though this was not observed. Notes were taken by the assistant during the focus groups to guide the ongoing conversation and support post‐session reflection and discussion but were excluded from the analysis, as the audio/video recordings provided more comprehensive and reliable data. This exclusion may have omitted some non‐verbal insights.

The sampling balanced shared and diverse experiences but relied on participants' willingness to share, possibly leading to selective disclosure. Trustworthiness was supported through careful sampling and systematic analysis. However, the interpretive nature of the study introduces subjectivity, potentially influencing result interpretation. To ensure rigour we used continuous analysis and comparison, strengthening credibility. Reflexivity was central, with ongoing reflection on our role as researchers and its influence on data collection and interpretation. By applying disciplined reflexivity and maintaining transparency, the analysis was thorough and credible. Although we followed S. E. Thorne's (2016) guidelines for quality and reflexivity, considering these limitations in the context of the study is important.

### 4.2 Recommendations for Further Research

Future studies may consider exploring confidential conversations with family members and next‐of‐kin. Understanding their experiences and how RNs can offer support could provide valuable insights into strengthening family involvement in palliative care.

Another area worth exploring is how RNs in high‐tech settings, such as ICUs or emergency departments, approach confidential conversations with patients at the end of life. These environments, often focused on life‐saving interventions, present unique challenges in establishing the trust and emotional connection.

It might also be helpful to explore how to create environments and structures that better support RNs in conducting confidential conversations in palliative care. Identifying aspects that help RNs manage these conversations could optimise care and improve the well‐being of RNs, patients and families.

Finally, it would be valuable to investigate how reflective practices and emotional resilience can be integrated into RNs' daily routines. Exploring how peer support, mentorship and ongoing learning can offer opportunities for RNs to further develop their conversational skills may help them navigate the emotional challenges of palliative care whilst fostering professional growth.

### 4.3 Implications for Policy and Practice

Our findings highlight the importance of reflection and emotional awareness in fostering confidential conversations whilst promoting RNs well‐being. Reflective practices in professional development can help RNs balance empathy with boundaries, build resilience and strengthen person‐centred care.

Healthcare institutions should prioritise environments that facilitate peer support, supervision and structured reflection. Encouraging a culture of open dialogue amongst colleagues strengthens well‐being, supports nurse retention and mitigates emotional exhaustion, ultimately contributing to sustainable nursing practice.

In nursing education, greater emphasis should be placed on reflection, self‐awareness and emotional resilience alongside clinical competencies. Training programmes that incorporate experiential learning, mentorship and peer discussion could better equip RNs to handle the complexities of confidential conversations.

By embedding reflective practices and peer support within healthcare culture, both caregiver and patient well‐being is improved whilst enhancing the overall quality of care. These strategies are relevant not only to palliative care but also across various healthcare settings to enhance both individual and collective resilience and to uphold the values of person‐centred care.

## 5 Conclusions

This study deepens the understanding of how RNs develop strategies to handle confidential conversations in palliative care. It highlights the role of reflective practices in strengthening resilience, enhancing communication and balancing empathy with professional boundaries. Using focus group interviews—an approach rarely applied in this context—this study uniquely captures the peer‐driven learning and emotional processing amongst RNs. Aligned with the principles of palliative care philosophy, the findings underscore the importance of structured peer support, supervision and continuous learning to sustain a resilient nursing workforce capable of delivering high‐quality, compassionate, person‐centred care.

Future research should expand on these findings by exploring how confidential conversations unfold in diverse healthcare settings and how best to equip RNs with the skills and resilience needed to manage them effectively. Strengthening these areas will not only enhance patient and family support but also contribute to a more sustainable and emotionally resilient nursing workforce.

## Author Contributions

T.S., Y.R., U.N., C.M.J.: Made substantial contributions to conception and design or acquisition of data or analysis and interpretation of data. T.S., Y.R., U.N., C.M.J.: Involved in drafting the manuscript or revising it critically for important intellectual content. T.S., Y.R., U.N., C.M.J.: Given final approval of the version to be published. Each author should have participated sufficiently in the work to take public responsibility for appropriate portions of the content. T.S., Y.R., U.N., C.M.J.: Agreed to be accountable for all aspects of the work in ensuring that questions related to the accuracy or integrity of any part of the work are appropriately investigated and resolved.

## Ethics Statement

The Swedish Ethical Review Authority, Regional Ethics Committee in Stockholm and Department of Other Research (Dns 2024‐00341‐02) approved the study. All RNs received written and verbal study information.

## Consent

RNs gave informed consent, understanding that participation was voluntary and withdrawal was possible at any time. The transcribed interviews were securely stored on a password‐protected computer, accessible only to the research team, ensuring ethical compliance. Quotes were translated as accurately as possible.

## Conflicts of Interest

The authors declare no conflicts of interest.

## Supporting information


Appendix S1.

Appendix S2.

Appendix S3.

Appendix S4.

## Data Availability

The datasets used and/or analysed during the current study are available from the corresponding author tove.stenman@miun.se under the prerequisite that no sensitive, personal or confidential data are revealed. The data and materials are stored on a password‐protected server at Mid Sweden University.
