# Supplementary material for: Beyond Everyday Small Talk: A Qualitative Study on Registered Nurses' Confidential Conversations in Palliative Care
Source: J Adv Nurs. 2025 Jun 3;82(2):1628–39. doi: 10.1111/jan.17098 (PMC12810652; doi:10.1111/jan.17098)
Supplement: Supplementary file 2 — Appendix S2. [file JAN-82-1628-s004.docx]

**Appendix (B) - Patient story**

On a bitterly cold February morning, I am scheduled to make a home visit to a woman I’ve never met before. She is affiliated with the specialist palliative home care team where I work. From the report I received beforehand, I learned that she has complex medical and nursing needs. She has refused assistance from municipal services, meaning I will be handling everything on my own, and I can expect the visit to take at least a couple of hours.

I’ve been informed that her attending physician at the home clinic, her oncologist, and the doctor overseeing the specialized palliative care team are all concerned. It has been impossible to have meaningful discussions with her about her current condition, the future, the possibility of ending oncological treatments, treatment strategies, or limitations. Every attempt to address her illness or the prospect of death has resulted in her becoming angry and either walking out of the meetings or throwing the doctors out. Even the unit’s social worker has tried to engage with her but without success.

I anticipate a long visit, and the care required will be demanding for both her and me. After several hours of administering injections, infusions, flushing lines, mixing drips, assisting with washing, and providing bed care, one last task remains: dressing a large, difficult wound. Throughout the visit, she makes small talk about everyday topics, like the weather, what she plans to cook, and their summer plans. She also reminisces about her past—her travels, work, and life experiences. I remain mostly quiet and focused on the tasks at hand.

The wound care is challenging, the bed cannot be raised so I am bent over her in an uncomfortable position. After several hours, I’m left feeling tired and sweaty. Suddenly, I notice she’s silent for a moment, gazing out the window at the magical beauty outside. Then, softly, she says:

“I know I’m going to die. It’s not that. It’s just that I don’t want to leave my children.”
