# Supplementary material for: Beyond Everyday Small Talk: A Qualitative Study on Registered Nurses' Confidential Conversations in Palliative Care
Source: J Adv Nurs. 2025 Jun 3;82(2):1628–39. doi: 10.1111/jan.17098 (PMC12810652; doi:10.1111/jan.17098)
Supplement: Supplementary file 3 — Appendix S3. [file JAN-82-1628-s003.docx]

**Appendix C – Conversation Guide**

Opening Question:

- You reviewed a patient case before coming here. Do you have any reflections on that case?

Introduction:

- These conversations are typically spontaneous and often occur alongside other nursing interventions. They tend to involve existential content.

Introductory Questions:

- What experiences have you had with confidential conversations?
- How do you feel during a confidential conversation?

Clarifying/Follow-up Question:

- When do you feel you’ve succeeded in connecting with a patient, physically, emotionally or spiritually?

Transition Questions:

- What opportunities do you see for engaging in confidential conversations?
- What obstacles do you face in conducting confidential conversations?

Key Question:

- What kind of support is needed to facilitate confidential conversations?

Clarifying/Follow-up Question:

- As a nurse, what do you need—whether knowledge, tools, or support—to effectively carry out confidential conversations? What would make these conversations easier for you?

Final Questions:

- How can we ensure that patients who want to talk are given the opportunity to do so?
- Is there anything else you would like to add before we conclude?
