# Supplementary material for: Beyond Everyday Small Talk: A Qualitative Study on Registered Nurses' Confidential Conversations in Palliative Care
Source: J Adv Nurs. 2025 Jun 3;82(2):1628–39. doi: 10.1111/jan.17098 (PMC12810652; doi:10.1111/jan.17098)
Supplement: Supplementary file 4 — Appendix S4. [file JAN-82-1628-s002.docx]

Appendix D-Examples of the analysis process

| Substantial sections of text | Key comments | Codes | Themes |
| --- | --- | --- | --- |
| Focus group 1  **104**: Then maybe you still gained their trust, but at that moment it was a bit chaotic, yet you can return to it because it is something you've earned, and I think that shows that I’ve listened to what you’ve said and reflected on it, so I would like to hear more.  **103**: Yes.  **104**: So, but it’s easy to… sometimes you have to bite your tongue, because when you’re stressed, it’s easy to respond with some cliché instead, like…  **103**: Exactly.  **102**: Yes, exactly, so it’s like… you know, just rush to say something, like: yes, that could be the case… that... No, but you know? Yeah, yeah.  **104**: It’s easily done. | Difficult to maintain trust when there is chaos.  By coming back, I want to show that I listened.  Easy to say a cliché when you're stressed. | Stress, chaos as obstacles  Coming back to confirm  Easy to make mistakes | Balancing external demands and inner motivation |
| Focus group 3:  **302**: Then I can imagine that it's also a bit different for us... for us as well... who we meet, just because you don’t always get that connection... That it becomes such an intimate conversation. Some don’t want to, and regardless of who is in front of you... some are... some find it easier to speak, and then it also depends a bit on how we are as... as those who are there.  **301**: Yes. I was going to say that too, sometimes I can really dive into things, and then I turn it around... I think, what was it that prevented me from getting there today? And then I can easily see that, well, today I was focused on a lot of other things at the same time, so maybe it shows... I’m not really, really, really there, even though I would like to be. That’s just how it is. We are only human... And... and sometimes I feel like I am very present, and that’s when many times they... they open up themselves. | We are different, and it's not always possible to establish the connection needed for intimate conversations.  After the patient meeting, I reflect and think about why I didn’t reach them.  If I’m not fully present, I can’t reach them.  We are only human. | Different people click in different ways  Not being able to reach someone  If I’m not fully present, I can’t reach them. I fail  We are only human | Balancing external demands and inner motivation |
| Focus group 3  **302**: Yes, then it's probably that... A confidential conversation... it means there is someone who listens. **301**: Yes... Yeah... **302**: It's not so important, what we say... what we say might not be the important thing, it's that we... they have someone who listens. **301**: A recipient... **302**: Without needing explanations for everything. | A confidential conversation is more about listening than about what is being said – being there as a recipient without always needing to provide answers or explanations. | To listen What we say is not important, it is listening that matters most Not giving explanations | Understanding and accepting personal limitations |
| Focus group 5  **504**: It's like, also, this thing we talk about a lot in supervision... which almost always comes up... that... this need to help and solve things and so on... in those moments, it's not about that, I mean... when I think about it... the opening is like... it's something shared, an acceptance, and how it may be more... Then you can go into problem-solving in other situations, but... not where this existential opening is... now I’m thinking out loud... **501**: Yes, but it feels pretty clear... those things are usually not things you fix in any way, it's just... they're things you just... **504**: No. **501**: Yes... this is just something I have to receive... **505**: And harbor it in a way... And that you also maybe have a little bit of the responsibility to not start problem-solving... **504**: Exactly. **505**: Because it can be quite easy. I mean, just stopping, and... being in this moment... | Obstacles: ... this need to help and solve. The opening happens in a shared space, an acceptance. Nothing we need to solve, nothing we need to fix. I just need to receive. And harbor it, the responsibility is not to solve the problem. But it's difficult. Stop and be present in the meeting. | Obstacles: my own need to help and solve. Nothing we need to solve, nothing we need to fix. Just receive, harbor it, the responsibility is not to solve the problem. Difficult. Presence/stop and be in the moment. | Understanding and accepting personal limitations |
| Focus group 5  **504**: Do you think that... when we had the first supervision, it became so clear that we needed comfort... it was almost unbearable for us to endure what she... **505**: ... had to endure... **504**: Yes. Yes, and then it was like, when the chaplain had noticed this in us... yes, then suddenly it was much easier to endure... I don't know... but just being validated that... we... I think we were... it was hopeless in the beginning. **505**: ... desperate... | Being in the unbearable made us need comfort.  The unbearable that we had to endure.  We were inconsolable from the beginning.  Desperate. | The unbearable Nurses need comfort Endure the unbearable Be desperate | Managing compassion with responsibility |
| Focus group 4  **403**: Yes… but also to dare, because you might break down yourself too… when it’s especially sensitive and touches you… like with patient xxx… for instance… I can imagine… it really affected me… **404** and **405**: Yes, yes, yes. **403**: And then it’s about daring to stay present, because otherwise… I might break down if it becomes too overwhelming now… and especially then with the relatives there, who were… well, when it… **405**: Yes, I agree. **404**: Yes. But I also think it’s OK for us to show emotions too… **403**: Absolutely. **404**: You… like… don’t have to be afraid of that… | It’s about daring to stay present and manage your own emotions, especially in sensitive situations that can be overwhelming. Showing your own emotions is also okay, and it’s important not to be afraid to express them, as it can strengthen human connection. | to dare to be moved to break down. to show emotions – to be affected. | Managing compassion with responsibility |
| Focus group 2  **202:** I think… at least for people who find it a bit difficult to open up, I think it might be better if it’s approached like that. **204:** Don’t force it… **201:** It feels like a threat when you look at them… like you’re demanding something. **203:** Do something else, but still maintain total presence. **201:** Yes, I had a hematology patient once… we played cards, and… after maybe three weeks, we started talking about the ‘white archive.’ Then, after four weeks, we filled it in together with her daughters. That’s when she decided she wanted to stay at home after all. She chose the music, shared stories about her life, and… well, it all started with the card game. **202:** Which ended with, yes, well… but that’s probably how you build up the whole thing… | Not forcing the conversation, taking it slow, and waiting for the patient. Otherwise, it can feel threatening.  Engaging in another activity with total presence as a facilitator.  Building the relationship and trust in a way that suits the individual patient, for example, by playing cards. | Not forcing the conversation, as it can feel threatening. Presence and other activities as facilitators. Building the relationship through person-centered care. | Gradual Engagement in Patient Trust-Building |
